# Proceedings of the Medical Association of the State of Alabama

**Published:** 1851-05

**Authors:** 


### ARTICLE IV.

*Proceedings of the Medical Association of the State of Alabama, begun and held in the city of Mobile, Dec.* 10-14, 1850. With an Appendix, pp. 156. octavo.

Dr. Lopez president called the meeting to order, when the usual business of such bodies was transacted.

The following is a list of the Officers elected for the ensuing year.

Dr. Chas. F. Lavender, of Selma President.
" Wm. O. Baldwin, of Montgomery, 1st Vice President;
" L. H. Anderson, Sumter, 2d Vice President ;
" Wm. B. Crawford, Mobile, 3d Vice President;
" A. Lopez, Mobile, Corresponding Secretary;
" Geo. A. Ketchum, Mobile, 1st Recording Secretary ;
" Jno. P. Barnes, Mobile 2d Recording Secretary ;
" H. M. Jackson, Montgomery, Treasurer ;
" W. H. Anderson, Mobile, Orator ;
" Chas. F. Percival, Church Hill Alternate.

Amongst many interesting resolutions adopted we observe the following :

Dr. Lopez offered the following resolutions :

*Resolved*, That a committee of —— be appointed, to draft a memorial, to be laid before the Legislature of the State, at its next regular session, setting forth the necessity, and advantages, which recommend the establishment of an Insane Asylum, and that said committee be requested to attend at Montgomery, for the purpose of aiding Miss Dix, in her efforts to that end.

*Resolved*, That, in pursuance of this object, the members of this Association, generally, be requested to ascertain, to every possible extent, the number, age, sex, color, and condition of Lunatics and Idiots within their respective counties, and obtain like information from those counties unrepresented, and forward the same to the chairman of the committee, on or before the 1st day of July next.

Prof. S. D. Gross, now of New York was elected an honorary member. A list of delegates was appointed to attend the American Medical Association.

The interesting reports, for want of room, cannot receive notice now. Take it alltogether the report shows an exceedingly good state of feeling and interest in the profession in Alabama.

